# Six years of grazing exclusion is the optimum duration in the alpine meadow-steppe of the north-eastern Qinghai-Tibetan Plateau

**DOI:** 10.1038/s41598-018-35273-y

**Published:** 2018-11-22

**Authors:** Wen Li, Yuzhen Liu, Jinlan Wang, Shangli Shi, Wenxia Cao

**Affiliations:** Grassland Ecosystem Key Laboratory of the Ministry of Education, Sino-U.S. Research Centres for Sustainable Grassland and Livestock Management, Grassland Science College of Gansu Agricultural University, Lanzhou, 730070 People’s Republic of China

## Abstract

Grazing exclusion is an effective management strategy for restoring degraded grasslands worldwide, but the effects of different exclusion durations on vegetation structure and soil properties remain unclear. Therefore, we evaluated vegetation characteristics and soil properties in an alpine meadow-steppe under grazing exclusion of different lengths (with grazing and with 3-year, 6-year, 9-year and 11-year grazing exclusions) on the Qinghai-Tibetan Plateau (QTP). We also explored the relationships among above-ground biomass, biodiversity and soil properties to ascertain the mechanism underlying the impact of grazing exclusion on these factors. The results showed that the above- and below-ground biomass, total number of plant species, community density, Shannon–Wiener diversity index, evenness index, richness index, soil and vegetation carbon (C) and nitrogen (N) storage and ecosystem C and N storage exhibited a hump-shaped pattern in response to the length of grazing exclusion with a 6-year threshold. In addition, structural equation modelling showed that the bulk density, soil moisture content, micro sand content and clay and silt contents were the most important determining factors leading to an increase in above-ground biomass in the alpine meadow-steppe after grazing exclusion, whereas the soil total N, available N, available phosphate and soil organic C content were the most important determining factors leading to a decrease in biodiversity. Considering the stability of the plant community and the C and N pools, long-term grazing exclusion (>9 years) is unnecessary, and the optimum exclosure duration of the moderately degraded *Elymus nutans - Kobresia humilis* type alpine meadow-steppe is six years on the north-eastern QTP.

## Introduction

As the largest terrestrial ecosystems, grasslands account for approximately 40% of the land surface of the earth^1^. Grasslands are not only a valuable base for livestock production but also perform significant ecological service functions^2–4^. However, approximately 90% of the grasslands in China have been degraded in recent decades^5,6^. In addition to affecting economic sustainability and development, grassland degradation threatens social stability and ecological security^7^. Therefore, grassland degradation has become a serious issue in China^7^, and how to restore degraded grasslands has simultaneously become a central scientific problem^7^. Depending on its capacity for self-recovery, a degraded grassland generally has the potential to regain its procreative and ecological functions if disturbances cease for an extended time^8^. Thus, the “Returning Grazing Land to Grassland” national ecological programme was implemented in 2003. Grazing exclusion using mesh fencing is considered the most effective management strategy to restore degraded grasslands worldwide^5,9–11^.

Previous studies involving grazing exclusion have mainly focused on vegetation structure^5,12–14^, soil properties^11,15,16^ and C and N cycles^17–19^ and have generally considered grazing exclusion to be an effective approach for restoring degraded grasslands. Li *et al*.^19^ focused on the effects of short-term grazing exclusion (4–5 years) on biodiversity and found that grazing exclusion significantly increased biodiversity compared with that in grazed grassland. However, Nishizawa *et al*.^15^ indicated that 11 years of grazing exclusion significantly decreased species richness and diversity in northeastern Hokkaido, Japan. Jing *et al*.^20^ observed that long-term grazing exclusion (30 years) decreased the biodiversity and biomass in a typical steppe grassland on the Loess Plateau, and due to excess litter accumulation, long-term grazing exclusion also inhibited grassland renewal^15,20^. Furthermore, time is a very important factor in ecological restoration^20^. According to the “dynamic disequilibrium” theory, a disturbance results in short-term temporal changes in the C pool but has no effect on long-term C dynamics^21^. Thus, the length of grazing exclusion plays an important role in shaping the trajectory of C dynamics^18^. Therefore, how long should grazing exclusion be applied to ensure optimal productivity and biodiversity? As the major producer in grassland ecosystems, the vegetation is a vital part of grassland structure and function^22^. To sustainably manage grassland ecosystems over the long term, it is essential to understand vegetation recovery dynamics in relation to soil properties following grazing exclusion.

Previous studies have focused on individual changes in soil or vegetation without exploring the impacts of grazing exclusion on the vegetation structure in relation to soil properties. Moreover, they have mainly focused on single exclusion durations and simply pair-compared grazed vs. un-grazed grasslands. Furthermore, the effects of different exclusion durations on the vegetation structure and soil properties remain unclear^23^. Therefore, we compared the vegetation, soil characteristics, and C and N storage under different grazing exclusion durations (0 years, 3 years, 6 years, 9 years and 11 years) in an alpine meadow-steppe of the QTP. The main purposes of this research were to (1) explore the optimal exclusion duration in the alpine meadow-steppe of the QTP, (2) understand the relationship between the vegetation structure and soil properties, (3) identify the factors affecting the vegetation structure, and (4) explore the mechanism underlying the effect of grazing exclusion on vegetation restoration dynamics in relation to soil properties. The final objective of this study was to provide new insights into the long-term sustainable management of grassland ecosystems in the alpine meadow-steppe of the QTP.

## Results

### Vegetation characteristics

We recorded a total of 26 species in the experimental area belonging to 24 genera and 13 families (Table 1). The numbers of plant species, genera and families exhibited a hump-shaped response pattern with increasing exclusion duration, with the threshold being the 6-year grazing exclusion plot (F6). The maximum numbers of species, genera, and families were found in the 6-year grazing exclusion plot (21 species), whereas the minimum numbers were observed in the CG plot (9 species). The plant species in the F9 and F11 plots decreased by 9 and 10 species, respectively, compared with the number in the F6 plot (Table 1).

**Table 1:** Species composition with different grazing exclusion durations on the Qinghai-Tibetan Plateau.

Species	Lifeform	Functional group	Importance value				
			CG	F3	F6	F9	F11
*Elymus nutans*	P	GG		0.607	0.853	0.964	0.984
*Poa crymophila*	P	GG		0.529	0.601	0.413	0.515
*Leymus secalinus*	P	GG		0.114	0.394	0309	0.479
*Koeleria cristata*	P	GG		0.238	0.281		0.102
*Stipa aliena*	P	GG		0.247			
*Bromus inermis*	P	GG				0.249	
*Kobresia humilis*	P	SG	0.557	0.311	0.385		
*Carex vulpina*	P	SG			0.462		
*Medicago ruthenia* var. *inschanica*	P	LG	0.081	0.211	0.152	0.252	0.179
*Artemisia smithii*	P	FG	0.192	0.334	0.214	0.187	0.146
*Thalictrum alpinum*	P	FG	0.053	0.085	0.115	0.062	0.076
*Potentilla bifurca*	P	FG		0.105	0.121	0.111	0.145
*Taraxacum mongolicum*	P	FG		0.152	0.202	0.207	0.238
*Allium sikkimense*	P	FG		0.301	0.314	0.284	0.315
*Oxytropis ochrocephala*	P	NG	0.104	0.101	0.188	0.180	
*Potentilla anserine*	P	FG	0.194	0.127	0.037		
*Polygonum viviparum*	P	FG			0.351	0.143	0.177
*Leontopodium nanum*	P	FG	0.091				
*Geranium pratense*	P	FG			0.235		
*Sphallerocarpus gracilis*	P	FG			0.127		
*Potentilla multifida*	P	FG			0.105		
*Heteropappus altaicus*	P	FG			0.155		
*Euphrasia pectinata*	A	FG			0.152		
*Gentiana straminea*	P	NG		0.107	0.221		
*Stellera chamaejasme*	P	NG	0.262				
*Achnatherum inebrians*	P	NG	0.628				
Total number (species)			9	15	21	12	11

CG, F3, F6, F9 and F11 represent the grazing and 3-year, 6-year, 9-year and 11-year grazing exclusion plots, respectively. For the types of species lifecycles, P represents perennials, and A represents annuals. The five functional groups were GG (grass species group), SG (sedge species group), LG (leguminous species group), FG (forb species group) and NG (noxious species group).

**Figure 1:**
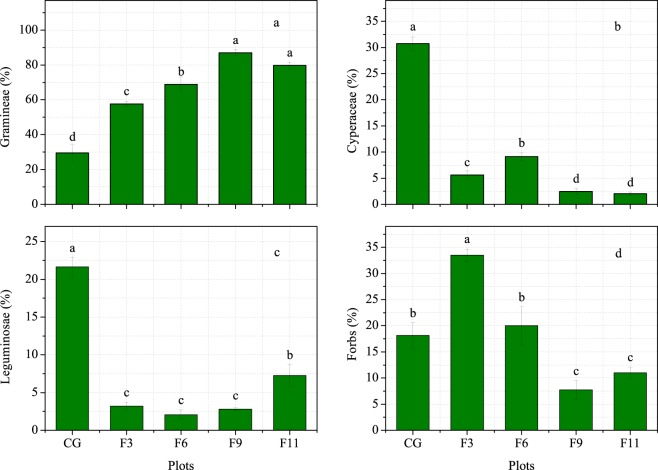
The effects of grazing exclusion of different durations on the Gramineae species group (**a**), Cyperaceae species group (**b**), Leguminosae species group (**c**) and forb species group (**d**) in an alpine meadow-steppe ecosystem on the Qinghai-Tibetan Plateau. CG, F3, F6, F9 and F11 represent the grazing and 3-year, 6-year, 9-year and 11-year grazing exclusion plots, respectively. Values are presented as the means ± standard errors. Different letters denote significant differences at *P* < 0.05.

Grazing exclusion significantly decreased the diversity and evenness indices compared with those of the CG plot, while the indices reached their peaks in the F6 plot after grazing exclusion. The maximum richness index was found in the F6 plot. The grazing exclusion plots all exhibited significantly increased plant density and coverage. The above- and below-ground biomass exhibited a hump-shaped pattern in response to the length of grazing exclusion, and the threshold was 6 years (Table 2). Compared with that in the CG plot, the above-ground biomass in the F3-F11 plots increased 4.6-, 9.0-, 8.3- and 5.6-fold, respectively, and the below-ground biomass increased by 81.3%, 162.3%, 136.4% and 136.2%. Grazing exclusion increased the proportion of Gramineae biomass but decreased that of Cyperaceae and Leguminosae. However, 3 years of grazing exclusion significantly increased the proportion of forb biomass, while 9 and 11 years of grazing exclusion significantly decreased the proportion of forb biomass (Fig. 1d). The litter biomass increased monotonically with the duration of grazing exclusion. Compared with that in the CG plot, the litter biomass in the F3-F11 plots increased 12.8-, 23.1-, 26.5- and 33.0-fold, respectively. All these values indicate that excluding the alpine meadow-steppe from grazing for 6 years not only significantly increased above-ground biomass but also maintained the plant community at a high level of biodiversity.

**Table 2:** The biomass, coverage, density and biodiversity with different grazing exclusion durations on the Qinghai-Tibetan Plateau.

Plots	Above-ground biomass (g·m^−2^)	Below-ground biomass (g·m^−2^)	Litter biomass (g·m^−2^)	Coverage (%)	Density (individual·m^−2^)	Shannon–Wiener diversity index (*H*)	Evenness index (*J*)	Richness index (*S*)
CG	62.06 ± 5.29c	2027.0 ± 69.31d	12.69 ± 0.42e	31.74 ± 1.44d	547.50 ± 11.21e	1.33 ± 0.02a	0.60 ± 0.01a	8.50 ± 0.43c
F3	349.02 ± 20.17b	3674.64 ± 71.67c	168.11 ± 5.99d	90.52 ± 3.18a	890.17 ± 14.5c	0.97 ± 0.01c	0.38 ± 0.01b	13.67 ± 0.76b
F6	619.55 ± 35.91a	5317.05 ± 128.53a	324.27 ± 6.21c	100.00 ± 0.00a	1340.00 ± 46.84a	1.13 ± 0.01b	0.39 ± 0.01b	18.67 ± 0.67a
F9	578.96 ± 9.07a	4792.10 ± 99.7b	349.53 ± 5.96b	100.00 ± 0.00a	960.50 ± 11.62b	0.76 ± 0.01d	0.36 ± 0.01c	9.33 ± 0.56c
F11	408.65 ± 12.55b	4786.91 ± 79.38b	447.52 ± 6.62a	100.00 ± 0.00a	763.50 ± 12.09d	0.74 ± 0.01d	0.32 ± 0.01d	9.00 ± 0.45c

CG, F3, F6, F9 and F11 represent the grazing and 3-year, 6-year, 9-year and 11-year grazing exclusion plots, respectively. Values are presented as the means ± standard errors. Different letters in the same column denote significant differences at *P* < 0.05.

### Soil properties

**Figure 2:**
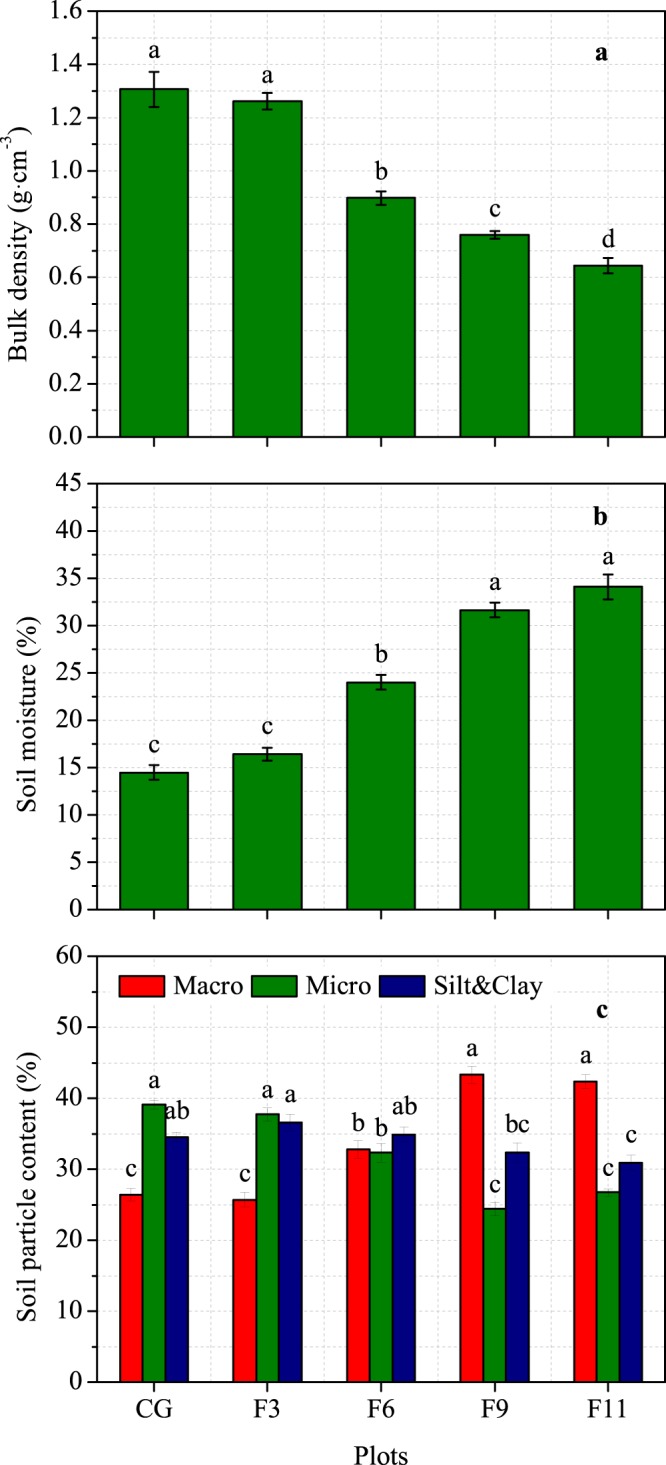
Soil bulk density (**a**), soil moisture (**b**) and soil particle content (**c**) at 0–10 cm depth in an alpine meadow-steppe ecosystem under different exclosure durations on the Qinghai-Tibetan Plateau. CG, F3, F6, F9 and F11 represent the grazing and 3-year, 6-year, 9-year and 11-year grazing exclusion plots, respectively. Values are presented as the means ± standard errors. Different letters denote significant differences at *P* < 0.05.

As shown in Fig. 2a, grazing exclusion significantly decreased the soil bulk density. Compared with that in CG, the bulk density in the F6, F9 and F11 plots decreased by 31.3%, 41.9% and 50.7%, respectively (Fig. 2a), but 6, 9 and 11 years of grazing exclusion significantly increased the soil moisture content (Fig. 2b). The soil moisture contents at the F6, F9 and F11 plots were 66.0%, 118.7% and 135.4% higher than that in the CG plot, respectively. Grazing exclusion significantly increased the macro-sand content, except in the F3 plot, but it significantly decreased the micro-sand content, except for in the F3 plot (Fig. 2c). The micro-sand content in the F6, F9 and F11 plots decreased by 17.3%, 37.6% and 31.6%, respectively, compared with that in the CG plot. Grazing exclusion had no significant effect on the silt and clay contents, except for in the F11 plot.

**Figure 3:**
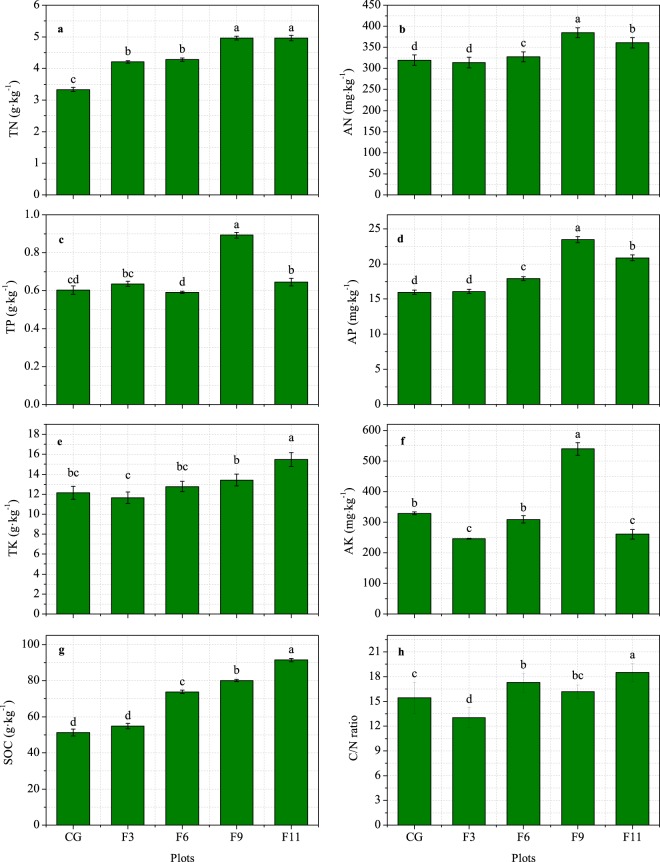
Changes in soil chemical properties at 0–10 cm depth in an alpine meadow-steppe ecosystem under different grazing exclusion durations on the Qinghai-Tibetan Plateau. CG, F3, F6, F9 and F11 represent the grazing and 3-year, 6-year, 9-year and 11-year grazing exclusion plots, respectively. TN, total nitrogen; TP, total phosphorus; TK, total potassium; SOC, soil organic carbon; AN, available nitrogen; AP, available phosphorus; AK, available potassium. Values are presented as the means ± standard errors. Different letters denote significant differences at *P* < 0.05.

As shown in Fig. 3, grazing exclusion had a significant effect on the soil nutrient contents. The soil total N, total potassium and soil organic C contents increased with grazing exclusion duration. Grazing exclusion significantly increased the soil total N content. The soil organic C exhibited the same trend as the total N content and significantly increased by 40.5%, 53.6% and 76.9% in the F6-F11 plots, respectively, compared with the level in the CG plot (Fig. 3g). Compared with the CG plot, only F9 and F11 exhibited significantly increased soil total phosphate. Compared with the CG plot, the F9 and F11 plots exhibited significantly increased available N content, by 20.3% and 12.8%, respectively. The highest soil available N, available phosphate and available potassium contents were found in the F9 plot, while F11 exhibited a significant increase in the C/N ratio.

### Carbon and nitrogen storage

**Figure 4:**
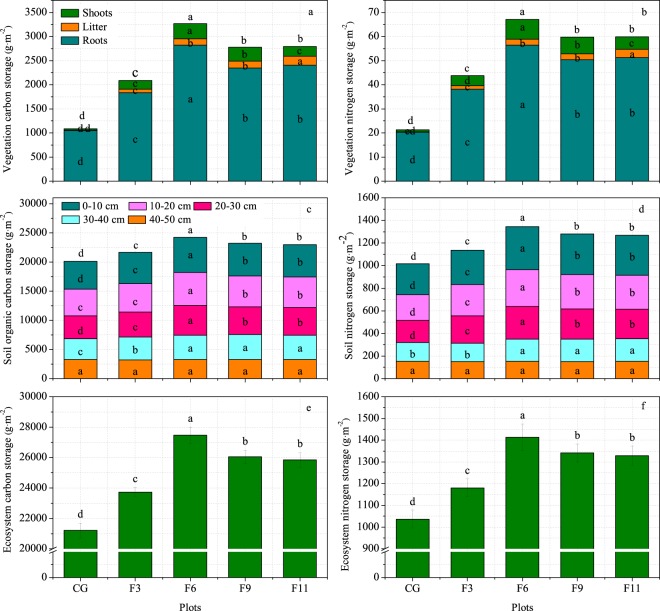
Changes in carbon and nitrogen storage in an alpine meadow-steppe ecosystem under different grazing exclusion durations on the Qinghai-Tibetan Plateau. CG, F3, F6, F9 and F11 represent the grazing and 3-year, 6-year, 9-year and 11-year grazing exclusion plots, respectively. Values are presented as the means ± standard errors. Different letters denote significant differences at *P* < 0.05.

As shown in Fig. 4, the vegetation, soil and ecosystem C and N storage all exhibited a hump-shaped pattern in response to the length of grazing exclusion with a threshold of 6 years. The 3-, 6-, 9- and 11-year grazing exclusion plots all exhibited significantly increased shoot, litter and root C and N storage (Fig. 4a,b). The total vegetation C storage in the F3-11 plots increased 0.9-, 2.0-, 1.6- and 1.6-fold compared with that in the CG plot, respectively, and the vegetation N storage increased 1.1-, 2.2-, 1.8- and 1.8-fold. The soil organic C and N storage values were significantly different at the 0–40 m soil depth under different exclosure durations; there was no significant effect in deeper soil (Fig. 4c,d). The soil organic C storage in the 0–50 cm soil layer increased by 7.5%, 20.4%, 15.4% and 14.2% in the F3–11 plots compared with that in the CG plot, respectively, and the 0–50-cm soil N storage in the F3-11 plots increased by 11.9%, 32.5%, 26.1% and 24.9%. Moreover, the highest ecosystem C and N storage values of 27469.9 ± 229.2 g m^−2^ and 1412.9 ± 60.2 g m^−2^, respectively, which were significantly higher than those in the other plots, were found in the 6-year grazing exclusion site (Fig. 4e,f). However, there was no significant difference between the 9- and 11-year grazing exclusion plots. The ecosystem C storage in the F3-11 plots significantly increased by 11.9%, 29.5%, 22.8% and 21.8%, respectively, compared with that in the CG plot, while the ecosystem N storage in the F3-11 plots increased by 13.8%, 36.2%, 29.3% and 28.1%.

### Relationships between soil characteristics and above-ground biomass and plant diversity

**Figure 5:**
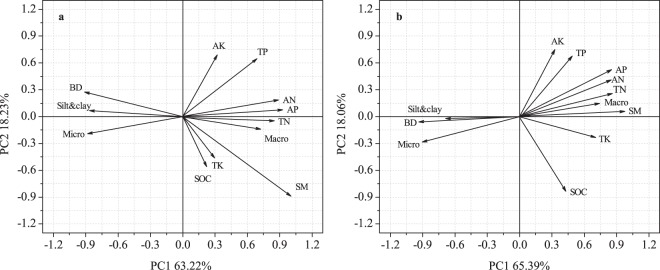
Principal component analyses (PCAs) of biomass and biodiversity as indicated by soil physicochemical properties. (**a**) Above-ground biomass, (**b**) biodiversity. TN- total nitrogen, TP- total phosphorus, TK- total potassium, AN- available nitrogen, AP- available phosphorus, AK- available potassium, SOM- soil organic matter, BD- bulk density, and SM- soil moisture.

We used principal component analysis (PCA) to determine the correlations among the above-ground biomass, biodiversity and soil physicochemical properties (Fig. 5). For the above-ground biomass, the first and second axes explained 63.22% and 18.23% of the standardized variance, respectively. The factors that have significant impact on above-ground biomass (loading value > 0.80) were the available N, micro-sand content, bulk density, available phosphorus, total N, silt and clay and soil moisture. Meanwhile, the first and second axes for biodiversity explained 65.39% and 18.06% of the standardized variance. The factors that have significant impact on biodiversity (loading value > 0.80) were available phosphorus, available N, total N, soil moisture, bulk density, macro-sand content, micro-sand content and soil organic carbon.

**Figure 6:**
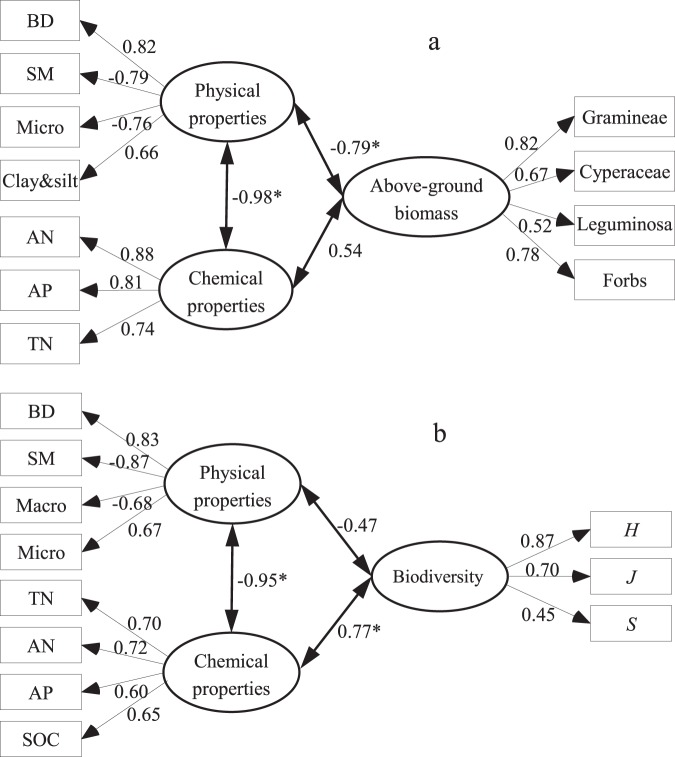
The structural equation model (SEM) of the direct and indirect effects of soil physicochemical properties on above-ground biomass **(a**) and biodiversity (**b**). *Represents significant differences at the 0.001 level. BD- bulk density, SM- soil moisture, AN- available nitrogen, AP- available phosphorus, TN- total nitrogen, SOC- soil organic carbon, *H*- Shannon–Wiener diversity index, *J*- evenness index, and *S*- richness index.

Based on the PCA results (Fig. 5), we used SEM analysis to explore the impact of soil physicochemical properties on above-ground biomass and biodiversity (Fig. 6), and the results explained the changes well (GFI was 0.847, χ^2^ = 16.17 and *P* = 0.19 for above-ground biomass; GFI was 0.805, χ^2^ = 10.06 and *P* = 0.27 for biodiversity). The SEM analyses showed that the soil properties and soil chemical properties directly altered the above-ground biomass and biodiversity. The soil physical properties pathway directly explained 62% of the total variance in above-ground biomass and 22% of the total variance in biodiversity. Similarly, the soil chemical properties pathway directly explained 29% of the total variance in above-ground biomass and 59% of the total variance in biodiversity. In addition, the effect of the chemical properties on above-ground biomass was not significant (*P* > 0.05), but the soil physical properties had a significant effect on above-ground biomass (*P* < 0.001). Inversely, the effect of the soil physical properties on biodiversity was not significant (*P* > 0.05), but the soil chemical properties had a significant positive effect on biodiversity (*P* < 0.001).

### Relationships between litter biomass and soil nutrients and plant diversity

**Figure 7:**
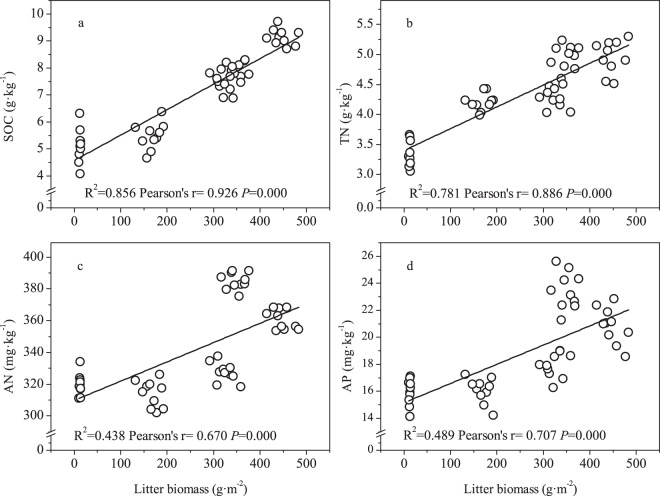
The relationships between litter biomass and SOC - soil organic carbon (**a**), TN - total nitrogen (**b**), AN - available nitrogen (**c**) and AP - available phosphorus (**d**).

**Figure 8:**
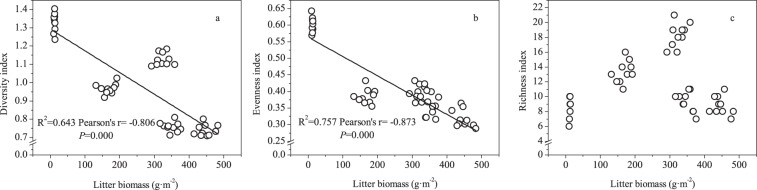
The relationships between litter biomass and the diversity index (**a**), evenness index (**b**) and richness index (**c**).

As shown in Fig. 7, the contents of soil organic C, total N, available N and available phosphorus in the 0–10 cm soil layer correlated strongly with litter biomass. The soil organic C, total N, available N and available phosphorus increased linearly with litter biomass (*P* < 0.01) (Fig. 7a–d). Meanwhile, the diversity and evenness indices negatively correlated with the litter biomass (*P* < 0.01) (Fig. 8a,b), but no relationship was found between litter biomass and the richness index (Fig. 8c).

## Discussion

Vegetation structure is an important aspect of evaluating ecosystem restoration^24^. Our results indicated that compared with the grazing treatment, grazing exclusion significantly increased the total number of species and plant density. This result was consistent with the results of Tang *et al*.^25^ and Kaouthar *et al*.^26^, who reported that grazing exclusion increased the number of species in the Horqin sandy land of China and in southern Tunisia, respectively. Grazing-induced plant mortality might decrease species diversity^5^. In the current research, graminoids, which are palatable to herbivores, had completely disappeared from the grazed plots due to overgrazing. Thus, the number of species decreased in the grazed plots. In addition, some species (*Potentilla bifurca*, *Taraxacum mongolicum* and *Allium sikkimense*) were too rare to be recorded in the grazed plots, whereas these species could become more abundant after a period of grazing exclusion. Fencing provides a suitable habitat for the establishment of a soil seed bank from which recruitment can occur^25^. However, the total number of species exhibited a hump-shaped pattern in response to the length of the grazing exclusion, with a threshold of 6 years. After 6 years of grazing exclusion, the alpine meadow-steppe on the QTP formed a suitable habitat for the growth of plant species; thus, both native and non-native species invaded the plot. Additionally, compared with the effects of the 6-year exclusion treatment, 9 and 10 species were lost from the 9- and 11-year exclusion plots, respectively. Ebrahimi *et al*.^23^ also reported that the maximum number of plants species was found in a 6-year grazing exclusion site in an arid rangeland of south-eastern Iran after grazing exclusion, and the reason may be greater competition for resources, such as light^27^ and/or nutrients^28^. In the current study, the graminoids, as the dominant species, were significantly more robust within the fenced plots, and the proportion of Gramineae biomass significantly increased with the exclusion duration. These large plants not only have a strong tillering ability but also occupy the upper canopy, which inhibits the growth of other plants. Therefore, some shorter plants in the lower layer of the community with weaker competitive ability diminished in density or even disappeared from the plant community due to the lack of light and/or nutrient availability^27,28^. In addition, the biodiversity indexes (Shannon-Wiener diversity index, evenness index and richness index) also showed an initially increasing and then decreasing trend with increased exclusion time, with a threshold of 6 years. Competitive dominance is a key factor regulating plant diversity^29^, and grazing exclusion generally had negative effects on biodiversity because it led to a reduction in plant density^5,30^. In our study, 11 years of grazing exclusion allowed the vegetation to be dominated by a few species (11 species) with strong colonization abilities. Hence, the plant biodiversity decreased. Therefore, considering plant community diversity and their community structure of different plant functional groups, the optimum exclosure duration is six years in the alpine meadow-steppe of the QTP.

Grazing accelerated above-ground biomass loss by herbivores and thus decreased the above-ground biomass^31^. Therefore, the remarkable improvement in pasture yield in the exclosure plots was directly related to decrease herbivore consumption, whereas the improvement in soil properties was beneficial to the restoration of the vegetation^14^. In the current study, grazing exclusion significantly increased above- and below-ground biomass and litter biomass compared with that in the grazing plots, and these results were consistent with previous studies^5,32,33^. However, the above- and below-ground biomass showed a hump-shaped response pattern with the length of grazing exclusion. The peak biomass was observed in the 6-year grazing exclusion plot, which may have been a result of plant species loss and a greater accumulation of litter biomass under the long-term fencing treatments. In our study, 10 species were lost in the 11-year grazing exclusion treatment compared with the 6-year grazing exclusion treatment, while the plant density decreased by 43% in the 11-year grazing exclusion treatment. Therefore, long-term fencing caused a surge of dominant *Elymus nutans* and simplified the vegetation community structure. Moreover, changes in species composition also significantly altered the above-ground biomass and litter biomass^34^. A dilemma exists between grazing utilization and the protection of the biodiversity of grasslands after grazing exclusion^5^. To resolve the contradiction between high productivity and low biodiversity after grazing exclusion, mowing or moderate periodic grazing should be conducted during the dormant stage in the alpine meadow-steppe ecosystems of the QTP. Therefore, considering the plant density, plant community productivity and utility value of herbivor grazing, the optimum exclosure duration is six years in the alpine meadow-steppe of the QTP.

Previous research has determined the changes in above-ground biomass and biodiversity after the removal of grazing pressure in this ecosystem^5,19,35,36^, but the mechanism underlying the effects of soil chemical and physical properties is poorly understood. Generally, the changes in soil chemical and physical properties are synchronous, but the property that plays the most important role in controlling the above-ground biomass and biodiversity is unclear. In the current study, the structural equation modelling analysis indicated that the soil physical properties were the critical determining factor leading to a decrease in above-ground biomass in the alpine meadow-steppe of the QTP after grazing exclusion, while the soil chemical properties were the critical determining factor leading to an increase in biodiversity after grazing exclusion. Grassland ecosystems are complicated; thus, the above-ground biomass and biodiversity are influenced by a series of factors, such as temperature, precipitation and topography. However, we did not include these factors in this research; therefore, the pattern of variation in above-ground biomass and biodiversity should be further explored in future research.

Generally, the negative effect of grazing on grasslands depends on trampling and browsing^5,37^. On the one hand, grazing can decrease the above- and below-ground biomass and litter biomass^38^, which reverts to soil after decomposition, and the litter decomposition rate is higher for palatable grasses than unpalatable grasses^39^. Grazing exclusion caused a surge of dominant *E*. *nutans*^19^, which is palatable to herbivores and thus accelerated litter decomposition. On the other hand, long-term trampling by herbivores can increase soil compaction and soil bulk density and thus decrease the activity of soil microorganisms and nutrient contents^40^. Conversely, grazing exclusion limits trampling by herbivores and improves the vegetation structure and soil physicochemical properties^19,41^, and the improvement of the soil physicochemical properties in the fenced meadow will have positive feedback effects on the structure of the plant community. In contrast, the ground surface of meadows subjected to grazing becomes barren because of continuous browsing and tramping, which leads to soil coarsening and soil nutrient loss^23^. In the current research, the grazing exclusion treatments significantly increased the soil nutrient content except at the site with 3 years of exclusion. The increase in soil organic C and total N concentration mainly results from greater organic matter (litter and dead roots) inputs^42^, and the contents of soil organic C, total N, available N and available phosphorus were positively correlated with litter biomass. Therefore, the increased litter biomass after grazing exclusion may be the main source of soil nutrients.

Grazing exclusion is an effective means of increasing C storage in a grassland ecosystem^18^. Our study showed that grazing exclusion significantly increased the vegetation C and N storage (in shoots, litter and roots), and the highest values were found in the site with 6 years of exclosure. These results were consistent with the results of other studies conducted in a semi-arid sagebrush steppe in Wyoming^43^ and may be due to the higher biomass of the exclosure sites. Meanwhile, grazing exclusion significantly increased C and N storage in the 0–50 cm soil layer, and the reasons for this may be as follows. First, the return of C from the above-ground biomass and litter biomass improved after grazing exclusion^42^. Litter decomposition is a main source of soil C input, and above-ground biomass may be returned to the soil when litter decomposes. Therefore, the above-ground biomass and litter biomass are the major drivers of soil C storage^44^. Second, root biomass is a vital factor in soil C storage^45^. In our study, grazing exclusion significantly increased the below-ground biomass (Table 2); thus, soil C storage may increase after grazing exclusion. Third, the variation in the dominant plants could influence soil C sequestration^44^. The joint presence of C4 grasses is a key cause of greater soil C accumulation in grassland ecosystems^46^. Therefore, the decrease in C3 grasses (*Kobresia humilis* and *Potentilla anserine*) and increase in C4 grasses (Cyperaceae spp. and Poaceae spp.) after grazing exclusion may explain the higher soil C accumulations in the exclosure soil than in the grazed soil. Fourth, vegetation recovery after grazing exclusion decreases C loss by wind erosion due to the greater plant coverage^47^. The C/N ratio is considered an important indicator in estimating the degree of the transformation of organic compounds in the soil^48^, and a higher C/N ratio indicates low decomposition of organic matter^49^. Our study indicated that 3 years of exclusion significantly decreased the C/N ratio, whereas 6, 9 and 11 years of exclusion all increased the C/N ratio. This result indicated that the decomposition rates were higher during the initial stage of fencing, whereas the decomposition rates were lower after 6 years of grazing exclusion than in the grazing site. Therefore, we concluded that the increase in soil C storage during the initial fencing stage may cause higher organic C input, whereas the increase in soil C storage after 6 years of fencing may be associated with a lower rate of organic C decomposition in the alpine meadow-steppe ecosystem of the QTP. This may be a result of short-term fencing having a beneficial effect on the diversity of soil microorganisms, whereas long-term fencing has a negative impact^15,50^. Meanwhile, C and N storage in the alpine meadow-steppe ecosystem exhibited a hump-shaped pattern in response to increased grazing exclusion duration, with a threshold of 6 years. This may have been caused by the higher C and N storage in the vegetation and soil at the site subject to 6 years of grazing exclusion. Therefore, considering the ecosystem C and N storage, the optimum exclosure duration is six years in the alpine meadow-steppe of the QTP, and this is enough to restore the ecological function of the grassland ecosystem for moderately degraded alpine meadows steppe.

## Conclusions

Grazing exclusion increased the above- and below-ground biomass, total number of plant species, community density, richness index, and C and N storage in the shoots, litter and roots but decreased the Shannon-Wiener diversity and evenness indices. Additionally, all these indexes showed a hump-shaped pattern in response to the length of grazing exclusion, with a threshold of 6 years. Long-term grazing exclusion in the alpine meadow-steppe of the QTP leads to lower plant biodiversity and density and results in the vegetation being dominated by a few species with strong colonization abilities. Thus, to solve this paradox of high productivity and low biodiversity after a fencing treatment, mowing or moderate grazing should be applied during the dormant stage. Furthermore, SEM showed that the bulk density, soil moisture content, micro-sand content and clay and silt contents were the critical determining factors leading to a decrease in above-ground biomass in the alpine meadow-steppe of the QTP after grazing exclusion, whereas the soil total N, available N, available phosphate and organic C content were the critical determining factors leading to an increase in biodiversity. Considering the stability of the plant community and the C and N pools, the optimum exclosure duration of the moderately degraded *Elymus nutans - Kobresia humilis* type alpine meadow-steppe is six years on the north-eastern QTP.

## Materials and Methods

### Site description

**Figure 9:**
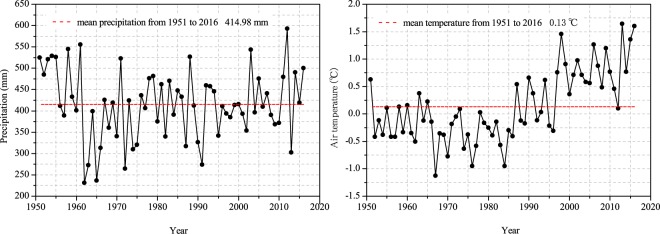
Changes in precipitation and temperature in the study area from 1951 to 2016 (climate data from Wushaoling Meteorological Station).

In this study, a field experiment was conducted in an alpine meadow-steppe region of the north-eastern QTP in Nannigou Village, Zhuaxixiulong Township, Tianzhu Tibetan Autonomous County, Gansu Province, PR China (37°11′N, 102°46′E; 2960 m a.s.l.). The study area has a typical Tibetan Plateau climate that is cold and wet for most of the year with thin air with low oxygen saturation levels, intense sunlight and high ultraviolet radiation. The long-term average annual temperature is 0.13 °C, varying from −11.4 °C in January to 11.2 °C in July, and the long-term mean annual precipitation is 414.98 mm, which mainly occurs from July to September (Fig. 9, climate data from Wushaoling Meteorological Station, 1951–2016). In this area, there is no absolute frost-free period throughout the year, and the length of the plant growing season is approximately 120 days, from May to September. The soil type is alpine chernozem, and the grassland type is alpine meadow-steppe. The vegetation is dominated by *Elymus nutans*, *Poa crymophila*, *Kobresia humilis*, *Koeleria cristata* and *Artemisia smithii*.

### Experimental design and field sampling

#### Experimental design

The grassland in the experimental area was moderately degraded (based on vegetation and soil characteristics) before 2003 according to the alpine meadow-steppe degradation criteria presented by Ma *et al*.^51^. Most of the degraded grassland in the experimental region had been fenced for livestock exclusion since 2003 because of the “Returning Grazing Land to Grassland” national ecological programme. In the current study, four sampling areas (approximately 11–13 ha per area) were randomly chosen across five different grazing exclusion durations (five treatments with four replicates each for a total of twenty plots. Ten sampling points were randomly established per plot), and all sampling areas were located at least 50 m away from each other. The treatments were four grazing exclusion treatments that had undergone succession for 3 (F3), 6 (F6), 9 (F9) and 11 (F11) years and grazed plots (CG, grazed all year at a livestock density of 7.0–8.0 head of Tibetan sheep per ha). The area of each fenced and grazed plot was 0.64 ha (80 m × 80 m). All plots were located 10–20 m away from each other. The five treatments were randomly assigned to each of the four sampling areas in a randomized complete block design. There were no differences in topography, soil type, and spatial heterogeneity among the experimental plots. The fenced sites were absolutely excluded from livestock grazing all year round.

#### Field sampling

A field survey was conducted in each of the fenced and grazed plots in early September of 2016 when the alpine plants reached their peak biomass. In each grazed and fenced plot, ten 1 m × 1 m quadrats were randomly established 1 m from the edge, and the plant species, coverage, height, and density of the respective species and the above- and below-ground and litter biomass were investigated in each quadrat. The above-ground biomass was cut at the soil surface by species, and all litter was collected in each quadrat. All the above-ground samples were immediately dried at 105 °C for 30 min and then oven-dried at 65 °C until a constant weight was achieved. After collecting the plants and litter, we used an auger (10 cm in inner diameter) to collect 0–60 cm soil samples (6 layers, 10 cm per layer) in each quadrat to measure the root biomass. Then, we separated the roots from the soil samples by washing the samples in a 0.5 mm mesh bag. The root samples were immediately dried for 30 min at 105 °C and then oven-dried at 65 °C until a constant weight was achieved. The soil bulk density (5 layers, 10 cm per layer) was measured by a cutting ring (5 cm in diameter and 5 cm high) in each harvested quadrat. Meanwhile, five random soil samples (0–10 cm in depth) were obtained by a 3.5 cm inner-diameter auger in each harvested quadrat and mixed into a single composite sample. The soil samples were air-dried to determine the physical and chemical properties.

#### Physical and chemical property analysis

The soil particle content was measured using a Malvern particle sizer (Mastersizer 2000, Malvern Corp., UK), and the soil moisture was measured using the oven drying method before air drying. The soil samples for chemical analysis were sieved through a 2 mm mesh sieve. The organic C content was measured using the dichromate oxidation method; the total N content was measured using the Kjeldahl method; the total phosphorus content was measured using the HClO_4_ - H_2_SO_4_ method; and the available phosphorus was measured using the molybdenum blue method. All these methods were performed according to Bao^52^.

#### Statistical analysis

The importance value was calculated using the methods of Lindsey^53^; the Shannon–Wiener diversity index (*H*), evenness index (*J*), and richness index (*S*) were calculated using the methods of Lindsey (1956); the C and N storage in the shoots was calculated using the methods of Fang *et al*.^54^; and the soil organic C and N storage was calculated using the methods of Guo *et al*.^55^. The ecosystem C and N storage was calculated as the sum of the C and N storage, respectively, of the shoots, litter, roots and soil. All data were analysed using SPSS software ver. 19.0 (SPSS for Windows, Version 19.0, Chicago, USA). We used a one-way ANOVA followed by Tukey’s multiple comparisons test at a significance level of *P* < 0.05 to compare the differences among the different exclosure durations. Considering the strong correlations among the above-ground biomass, productivity, biological density, and several soil physicochemical properties, we used a principal component analysis (PCA) to confirm the primary axes of covariation among the variables. Based on the PCA results, we used structural equation modelling (SEM) with above-ground biomass and density, soil physical properties and soil chemical properties as variables to identify the direct and indirect effect pathways; SEM analyses were conducted using Amos software ver. 21.0. We also used linear regression to explore the relationships between litter biomass and soil nutrient and biodiversity using Origin software, ver. 8.5.
